# Biogenic synthesis of silver nanoparticles using *Sida cuneifolia* leaf extract for enhanced antibacterial, cytotoxic, and anti-biofilm activities

**DOI:** 10.1016/j.biotno.2025.07.003

**Published:** 2025-07-28

**Authors:** Motasim Ismael, Madivoli Edwin, Khayeli Juliah

**Affiliations:** a Department of Molecular Biology and Biotechnology, Pan African University Institute for Basic Sciences Technology and Innovation (PAUSTI), P. O. Box 62000-00200, Nairobi, Kenya; b Department of Chemistry, Jomo Kenyatta University of Agriculture and Technology, P.O. Box 62000-00200, Nairobi, Kenya; c Department of Zoology, Jomo Kenyatta University of Agriculture and Technology, P.O. Box 62000-00200, Nairobi, Kenya; d Department of Botany, University of Nyala, P.O. Box 155, Nyala, South Darfur, Sudan

**Keywords:** AMR, *S*. *cuneifolia*, AgNPs, Antibacterial, Anti-Biofilm, Hemolysis, SEM

## Abstract

Antimicrobial resistance (AMR) is one of the global threats that needs to be addressed. Nanotechnology represents a promising way to address this issue due to its multifaceted mode of action. This study aimed to synthesize and evaluate the antimicrobial and anti-biofilm properties of silver nanoparticles using *S*. *cuneifolia* leaves extract. The formation and properties of AgNPs were characterized using a UV–Vis spectrophotometer, an FT-IR spectrophotometer, TEM, and XRD. Disc diffusion and MIC were used to evaluate the antibacterial activity of AgNPs towards *E. coli*, *S. flexneri*, and *S. aureus*. The antibacterial action of silver NPs was observed using SEM, and cytotoxicity was assessed using the hemolysis assay. The anti-biofilm was evaluated against *E. coli* and *S. aureus*. From the results obtained, a sharp peak in the UV–Vis spectra centered at 419 nm was associated with AgNPs, while the sharp, distinct peaks in the powder diffractograms were linked to the face-centered cubic (fcc) of crystalline AgNPs. TEM micrographs confirmed their spherical morphology, with dimensions varying from 4 to 31 nm. The nanoparticles showed significant antibacterial and anti-biofilm activities against the tested isolates. Additionally, SEM confirmed that they could destroy the cell membrane and cause death. The biocompatibility of the synthesized AgNPs was safe at 100 μg/mL. Therefore, *S. cuneifolia* leaf extract has the potential to be an environmentally friendly substitute for the fabrication of Ag nanoparticles. The findings reveal that the synthesized nanoparticles could serve as a secure and effective alternative for addressing AMR.

## Graphical abstract

**Figure:**
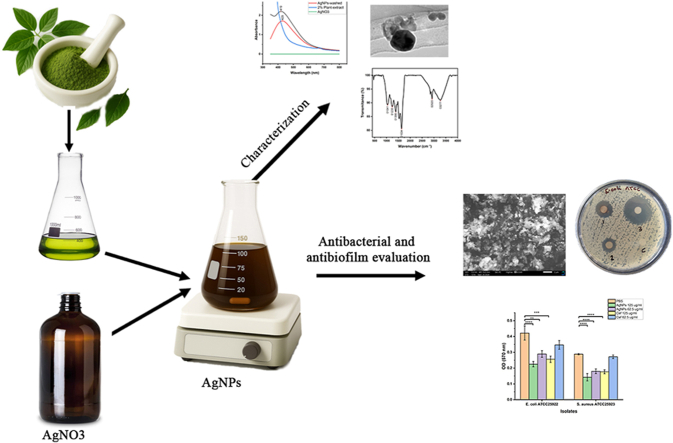

## 1 Introduction

The inappropriate use of antimicrobials in various domains, including human healthcare, animal healthcare, the environment, public health, and agriculture, has contributed to the development of antimicrobial resistance (AMR) worldwide.^1^ Furthermore, this resistance is attributed to the failure of bacterial infection treatment, which has become a global issue. The consequences of these resistant strains lead to challenging infection control, which ultimately results in prolonged treatments, increased healthcare expenses, and higher mortality rates.^2^, ^3^, ^4^ The statistics showed that in 2019, bacterial AMR contributed to the worldwide deaths of nearly five million,^1^, ^5^ and new therapeutic techniques are needed to mitigate this situation, especially those having novel mechanisms of action.^6^ Biofilms are known as self-synthesized extracellular substance (EPS) matrices consisting of different materials, including polysaccharides, proteins, and extracellular DNA (eDNA), which envelope multicellular bacterial populations, functioning as a physical barrier against the host immunological response and drugs.^7^ Thus, the bacteria that can form biofilms indicate their ability to resist antimicrobial therapies, which makes biofilm-related disorders exceedingly tough to handle.^6^, ^8^

Nanotechnology represents an attractive solution to address AMR, with Ag nanoparticles known for their effective and extensive antibacterial^9^ and anti-biofilm properties.^10^ The silver nanoparticles kill the bacteria in several ways, including embedding themselves into the bacterial cell membrane and disrupting it, forming reactive oxygen species that are toxic to bacteria, and also inactivating functions of some essential enzymes, which finally lead to the death of the bacteria.^10^, ^11^, ^12^ Furthermore, due to their effectiveness against both Gram-positive and Gram-negative (susceptible and resistant) bacteria and the difficulties in treating multidrug-resistant (MDR) pathogens with availably current antibiotics in addition to high cost of drug development, the nanotechnology and Ag nanoparticles highlight their promise as a multifaceted antibacterial treatment^13^; especially the ones having the ability to form a biofilm.^14^

Traditional methods of synthesizing Ag nanoparticles sometimes utilize hazardous chemicals and require substantial energy. Consequently, methods of synthesizing Ag nanoparticles often employ hazardous chemicals and require substantial energy, thereby limiting their practical applicability. Green synthesis, employing biological agents provides such as phytochemicals from plant extracts, offers an environmentally benign and sustainable alternative.^15^ Plant-based synthesis utilizes the bioactive chemicals included in plant extracts to facilitate nanoparticle production, hence improving their stability, biocompatibility, and antibacterial effectiveness.^9^ *S*. *cuneifolia* is a medicinal plantthat is used traditionally to treat several ailments caused by Enterobacteriaceae and *Staphylococcus* spp for its antibacterial characteristics^16^ and is a good option for the green synthesis of silver nanoparticles (AgNPs).

This study aims to inspect the environmentally sustainable synthesis of silver NPs utilizing the extract of *S. cuneifolia* leaves and their bactericidal and bacterial biofilm destruction evaluation. The findings reveal that the synthesized nanoparticles exhibit significant effectiveness against both sensitive and resistant isolates of Gram-positive and -negative bacteria; they may serve as a secure and effective alternative to conventional existing antimicrobials, addressing both antibiotic resistance disorders sustainably.

## 2 Materials and methods

### 2.1 Extraction

The *S. cuneifolia* plant was collected from Kiambu County (Kenya) (rain season), the plant has been classified and documented by Mr. John Kamau Muchuku at at Jomo Kenyata University of Agriculture and Technology Botanical Herbarium (JKUATBH) and with assigned reference MNMI-JKUAT/001/2024. The leaves were separated carefully and cleaned using distilled water (DW) to eliminate particles of dust and impurities. Then, the leaves were allowed to dry at ambient temperature in the shade for two weeks and ground into fine powder using a blender. A 2 % (w/v) solution was obtained by dispersing 4 g of ground powder in 200 mL distilled water; subsequently, the mixture was heated to 100 °C for 10 min and then allowed to cool to room temperature, and filtration was conducted to acquire the filtrate, which was subsequently stored at 4 °C before synthesis.^17^ Furthermore, the extract alone was freeze-dried and stored for its activity evaluation.

### 2.2 Synthesis of silver nanoparticles

A 1 mM silver nitrate (AgNO_3_) solution was prepared and heated to 70 °C under vigorous and continuous stirring. Subsequently, dropwise additions of 2 % of the aqueous extract were made in a 1:9 extract-to-silver nitrate ratio.^18^ The mixture was maintained under these conditions for 4 h. A transition in hue from pale yellow to brown was noted, signifying the synthesis of silver NPs. The solution underwent centrifugation at 5000 rpm for a duration of 20 min, washed twice, and dried using a freeze-dryer.^19^ To determine the optimum time for AgNP synthesis using *S. cuneifolia* leaf extract, the solution's absorbance was monitored at zero-minute and 1-h intervals for 4.5 h by a UV–vis 1800 spectrophotometer (Shimadzu, Japan) with adjusted wavelength from 200 to 800 nm.^20^ The synthesis of AgNPs was further confirmed by UV–vis spectroscopy. The 2 % extract and silver nitrate were employed as negative controls.^21^, ^22^

### 2.3 Characterization of AgNPs

The aqueous leaf extract of *S. cuneifolia* reduces silver ions and acts as a capping agent, ensuring the bio-reduced silver nanoparticles are stable. The functional groups in the silver nanoparticles (AgNPs) were examined utilizing an FT-IR-8000 spectrophotometer (Shimadzu, Japan). The scanning range was established between 400 and 4000 cm^−1^, and the spectral resolution was fixed at 4 cm^−1^. Subsequently, the peaks were identified and characterized accordingly.^18^

### 2.4 X-ray diffraction XRD and transmission electron microscope (TEM) analysis

Synthesized AgNPs were collected at 5000 rpm for 20 min and subsequently washed thrice with DW; then, the pellet was suspended in DW and used for XRD and TEM analysis. For XRD, the sample was dried on a standard XRD holder and analyzed using Cu Kα radiation (λ = 1.5418) by ULTIMA IV X-ray diffractometer (Rigaku, Japan). The angle measurement was adjusted from 2 to 70 theta degrees, with a speed of 2 and a step of 0.02°. Finally, the results of the AgNPs were analyzed and compared with those of the Crystallography Open Database (COD).^23^ JEM-2100 TEM (JEOL, Japan) at a voltage of 25 kV was used to analyze the nanoparticles.^18^

### 2.5 Minimum inhibitory concentration (MIC)

A 100 mg/mL stock solution of the nanoparticles was prepared by solubilizing the AgNPs in absolute dimethyl-sulfoxide (DMSO), which was kept at 4 °C for later use. A sterilized Müeller-Hinton broth (MHB) was used to dilute the stock solution to the desired treatment concentration (500 μg/mL), in which the DMSO concentration decreased drastically to 0.5 % (v/v). The MIC was determined against both Gram-positive, including *S. aureus,* both ATCC25923 and a resistant (R) clinical isolate, and Gram-negative, including *E. coli* ATCC25922 and a resistant clinical isolate, and *S. flexneri* ATCC12522 bacterial pathogens. A modified broth microdilution method of the National Committee for Clinical Laboratory Standards (NCCLS) was employed to ascertain the MIC as described in a previous study.^24^ One millilitre of MHB was inoculated with an entire loop of bacteria and incubated at 37 °C for 14 h, after which the suspensions were diluted to 5 × 10^5^ CFU/mL 100 μL of two-fold serially diluted AgNPs, with concentrations ranging from 0.97 to 500 μg/mL, were introduced into the wells, except for columns 11 and 12 of 96-well plates, followed by 100 μL of bacterial suspensions. Cultures without AgNPs and media only were set as a positive control, while the medium alone functioned as a negative control for sterility. Also, the positive control antibiotics, including ceftriaxone, amikacin, and vancomycin, which were purchased from the local supplier (LEGACY LAB AFRICA Ltd., Kenya) with a starting concentration of 250 μg/mL and plant extract with a starting concentration of 32 mg/mL, were tested alongside nanoparticles. The plates underwent incubation for 18 h at 37 °C, after which the turbidity was observed since the MIC has been defined as the minimum amount of AgNPs that did not allow observable turbidity. The MIC was performed in triplicate. Ten microliters from each well that showed no turbidity were applied to the Tryptic Soy Agar (TSA) plate surface and incubated for 24 h at 37 °C. The formation of bacterial colonies was observed to determine the MBC.

### 2.6 Disc diffusion assay

The effectiveness of the synthesized AgNPs using *S. cuneifolia* leaf extract against *S. aureus* ATCC25923, *S. aureus* (R), *E. coli* ATCC25922, *E. coli* (R), and *S. flexneri* ATCC12522 was assessed by the disc diffusion method, as in the previous study,^25^ these strains were selected as Gram-positive and -negative models (susceptible and resistant). Briefly, the bacterial suspensions at the mid-log phase in MHB were diluted to 0.5 McFarland standard. Subsequently, it was disseminated throughout the surface of Müeller-Hinton agar (MHA) plates utilizing sterile cotton swabs, followed by placing the discs (6 mm) on the surfaces. A 20 μL with the concentrations of 2.5 and 5 μg silver nanoparticles per disc, 100 μg/mL Ceftriaxone, and PBS was introduced into the discs, and the plates were maintained under standard conditions overnight. Zones of inhibition were observed and measured. Both positive (+ve) and negative (−ve) controls were ceftriaxone and PBS. The inhibitory zone's diameter, including the disc (6 mm), was measured in millimeters. To ensure visual consistency, the complete diameter was included in the graphs. The test was performed in triplicate.

### 2.7 Mode of action

A JCM-7000 NeoScope™ SEM (JEOL, Japan) was used to examine the impact of AgNPs on morphological changes of *S. aureus* (R), *E. coli* ATCC25922, *E. coli* (R), and *S. flexneri* ATCC12522 at different concentrations, 2× and 4× MIC. 200 μL of bacterial solution from a mid-log phase culture in MHB exhibiting a 1.5 × 10^8^ CFU/mL, was combined with an equal volume of 2× and 4× MIC concentrations of AgNPs or PBS. After the incubation for an hour at 37 °C, the mixtures were centrifuged at 4000×*g* for 10 min. Then, the pellets of the bacteria were washed thrice using PBS. 2.5 % glutaraldehyde in PBS was used to fix the bacteria at 4 °C overnight. The bacterial pellets were obtained through centrifugation (4000×*g* for 10 min) followed by dehydration using different ethanol concentration (30 %, 50 %, 70 %, 90 %, and 100 %) with an incubation time of 15 min. The samples were air-dried overnight at room temperature and spread on carbon tape, followed by SEM imaging.^26^

### 2.8 Hemolysis

Fresh whole sheep blood was obtained from the Zoology Department of Jomo Kenyatta University of Agriculture and Technology (JKUAT) (Ethical approval No. MKU/ISERC/4066). It wasapplied as a blood model. 1 mL of sheep erythrocytes underwent centrifugation for 5 min at 2000 rpm, after which the supernatant was removed. The pellet was washed thrice until the supernatant appeared colorless. 10 mL of PBS was used to resuspend the red blood cells (RBCs) (final concentration 10 %). 100 μL of erythrocyte suspension was incubated with an equal volume of the AgNPs in PBS (final concentration ranging from 25 to 800 μg/mL; two-fold serial dilution) at 37 °C for 2 h. Subsequently, centrifugation was performed for 5 min at 2000 rpm, after which 150 μL of supernatant was transferred to a 96-well plate. The plate was read by the microplate reader (BIOTEK elx800, USA) with OD adjusted to 540 nm. PBS and 1 % Triton X-100 were negative and positive controls, respectively.^27^ The equation utilized for calculating the hemolysis ratio is as follows:

$$Hemolysis\;\%=\frac{{OD}_{AgNPs}-{OD}_{PBS}}{{OD}_{Triton\;x}-{OD}_{PBS}}\times 100\%$$

### 2.9 Anti-biofilm assessment

The semi-quantitative analysis of crystal violet was employed to evaluate the impact of the produced nanoparticles on the bacterial biofilm, as described by Ismael.^24^ After seeding 96-well plates with the *E. coli* ATCC25922 and *S. aureus* ATCC25923 suspensions at a final concentration of 5 × 10^7^ CFU/mL in Tryptic Soy broth (TSB) enhanced with 1 % glucose, the plates were incubated for 48 h at 37 °C. Following the time of incubation, the free-floating cells and TSB media were taken out. PBS was used to wash the formed biofilms at the wells' bottom thrice, and the resulting biofilms were fixed with Neutral Buffered Formalin (NBF) (100 μL per well) for 20 min at ambient temperature; after the time of incubation, the fixed biofilms were washed at least 3 times to remove excess NBF, followed by treatment with 100 μL AgNPs of various concentrations (62.5 and 125 μg/mL) for 12 h at ambient temperature. Subsequently, the residual biofilms were subjected to staining with 20 μL of 1 % crystal violet for a duration of 15 min, then washed thrice with sterilized DW. The crystal violets incorporated at the well's bottom with the biofilms were dissolved in 95 % ethanol (200) μL. Following a 15-min incubation period at room temperature, 150 μL of the supernatants were transferred to a fresh 96-well plate and measured at OD 570 nm using a microplate reader (BIOTEK elx800, USA). PBS-treated biofilms served as the negative control, while ceftriaxone was used as the positive control. The test was performed in triplicate.

### 2.10 Long-term storage antimicrobial evaluation of the AgNPs

The long-term storage effect of the silver nanoparticles in suspension or colloidal form on the antimicrobial properties was extensively investigated,^28^, ^29^, ^30^ but not in lyophilized form. In this study, the main stock of the AgNPs was kept in a powder form in the dark at room temperature, and the stock solution was prepared and stored at 4 °C to be used within two weeks. After 252 days (8 months plus), a new stock solution was prepared from a lyophilized AgNPs and tested against the bacterial isolates using disc diffusion assay as mentioned in section 2.6; the test was performed in triplicate.

### 2.11 Graphing and statistical analysis

OriginPro® (2024b) software was used for graphing and data analysis, and every experiment was conducted a minimum of three times. Data were presented as the mean ± standard deviation and evaluated utilizing one-way analysis of variance (ANOVA) employing the Tukey test. Differences among several groups at ∗p < 0.05, ∗∗p < 0.01, ∗∗∗p < 0.001, ∗∗∗∗p < 0.0001 were deemed statistically significant.

## 3 Results and discussion

### 3.1 Visual and UV–vis analysis of synthesized AgNPs

**Fig. 1:**
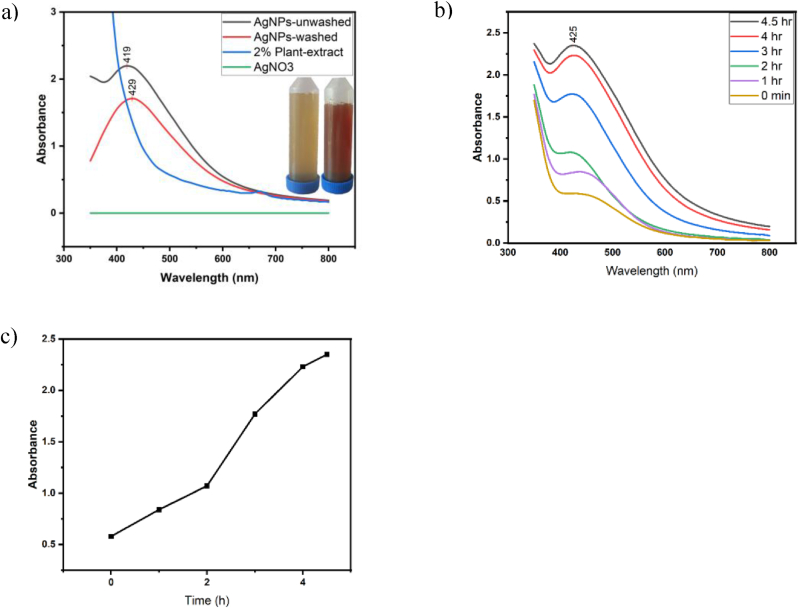
Characterization of *S. cuneifolia* leaf extract-mediated AgNPs. (a) UV–vis spectrum of synthesized AgNPs (unwashed and washed) and negative controls (2 % leaf extract and AgNO_3_); the inset is digital images of the extract and AgNPs solution. (b) UV–vis spectrum of synthesized AgNPs at different times (0, 1, 2, 3, 4, and 4.5 h). (c) Quantification analysis of B.

The transition in the mixture's color from light yellowish to brown indicated the successful creation of AgNPs in the aqueous extract of *S. cuneifolia*, Fig. 1(a). The analysis of UV–vis spectroscopy for AgNPs synthesized using *S*. *cuneifolia* leaf extract reveals distinct surface plasmon resonance (SPR) characteristics indicative of effective nanoparticle synthesis. The UV–vis absorbance of the synthesized nanoparticles before washing show a significant SPR peak at 419 nm (Fig. 1(a), confirming the formation of Ag nanoparticles with a substantial concentration. In contrast, the SPR peak of a washed sample of the AgNPs has shifted slightly to 429 nm Fig. 1(a) which indicates size changes, thus signifying alterations of the synthesized Ag nanoparticles milieu from eliminating the extra plant residues in the solution or other contaminants.^31^ Additionally, the pronounced absorbance of the synthesized AgNPs before washing indicates a greater extent of NPs concentration or aggregation than that of nanoparticles before washing.^32^ Furthermore, the decrease and minor shift in the absorbance peak of the sample after washing Fig. 1(a), indicate greater stability and a distributed AgNPs solution.^33^ Both 2 % of the plant extract and AgNO3 were applied as negative controls; the result showed that neither showed any significant absorbance at the silver nanoparticles region Fig. 1 (a), further confirming the findings. This finding is comparable to the finding of K. Riazunnisa, which used leaf extract for biosynthesis of AgNPs.^34^ The current investigation did not directly evaluate environmental toxicity; however, the production method utilized—incorporating *S. cuneifolia* extract as an agent for reduction and stabilizing reduces the reliance on harmful chemical substances typically associated with traditional NPs fabrication (such as sodium borohydride, hydrazine, or organic solvents).^35^ Green methods for fabrication are typically seen as more environmentally friendly due to the utilization of plant-based biological molecules, demand less energy, and produce fewer harmful byproducts.

The impact of time on silver nanoparticle formation utilizing *S. cuneifolia* leaf extract was examined via UV–vis spectroscopy. Absorbance spectra were documented across a 200–800 nm wavelength range at multiple time intervals (0 min, 1, 2, 3, 4, and 4.5 h). The absorbance peak at 425 nm was shown by UV–vis Fig. 1(b), signifying Ag nanoparticles formation. The UV–vis spectroscopy results show a negligible absorbance at the wavelength of 425 nm at zero minute, signifying minimum formation of the nanoparticles. As the synthesis reaction continued, the absorption value at this wavelength gradually rose, indicating a rise in silver NPs concentration. As shown in Fig. 1(c), beginning at 1 h onward, the absorbance exhibited a consistent increase, signifying the ongoing formation of silver NPs. The absorption value was roughly 0.5 at the beginning of the reaction, which increased steadily to over 2 after 4 h. After 4.5 h, the rate of increment began to level out, indicating that the reaction of the synthesis was either nearing completion or stabilizing. Finally, this finding concludes that the optimum time to synthesize AgNPs using *S. cuneifolia* leaf extract is 4 h.

### 3.2 FTIR analysis

**Fig. 2:**
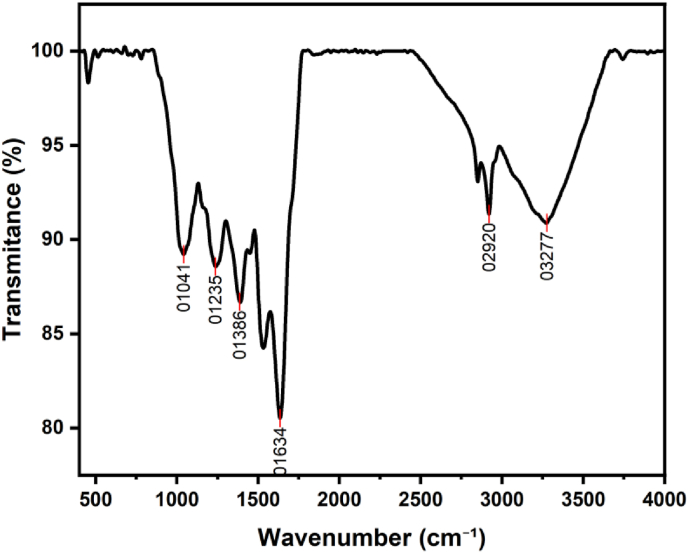
FTIR spectra of synthesized AgNPs utilizing extract from *S*. *cuneifolia*.

Fig. 2. The functional group present in AgNPs prepared using *S. cuneifolia* is depicted below, showing a wide range of absorption peaks, including 3277, 2920, 1634, 1386, 1235, and 1041 cm^−1^ (Fig. 2). These peaks correspond to vibrations of O–H, C–H, C=O, C–N, C–H, C–N stretching, and C–O stretching Table 1, respectively, and indicating different phytochemical compounds involved in the formation of the AgNPs, including phenolic compounds, alkaloids, proteins, amino acids, vitamins, and enzymes.^25^, ^36^, ^37^ Peaks at 3268–3432 cm^−1^ may signify the stretching of hydrogen-bonded hydroxyl groups (O–H) present in alcohols, phenolic, or glycosides.^38^ This suggests that the extract contains O–H-containing compounds, probably phenolics, which may contribute to the stability and reduction of Ag^+^ to AgNPs. Additionally, the peaks at 2930–2880 cm^−1^ indicate the alkanes' C–H stretching vibrations.^22^, ^39^, ^40^ Furthermore, 1617, 1626, and 1653 cm^−1^ peaks are associated with aldehyde or ketone (C=O) functional groups in flavonoid and tannin derivatives^21^, ^36^, ^41^ or possibly attributed to vibration of proteins' amide I, typically ranging from 1600 to 1700 cm^−1^.^38^, ^42^ Peaks nearly at 1634 cm^−1^ were reported in green synthesized AgNPs using *Jatropha gossypifolia*^38^*;* it might also point to C=C stretching vibrations in biological double-bond molecules' hydrocarbon segments, while the peak at 1041 cm^−1^ may correspond to C–O stretching.^40^ Clear peaks observed in this study may suggest that the leaf extract from *S. cuneifolia* contains organic substances, such as proteins, glycosides, or phenolics, that could act as reducing, capping, and stabilizing agents for forming AgNPs.

**Table 1:** FTIR peak positions and their correspondence to stretching vibrations.

Peak position (cm^−1^)	Assignment
3277	O–H
2920	C–H
1634	CO
1386	C–N
1235	C–H
1041	C–O

### 3.3 X-ray diffraction (XRD) analysis

**Fig. 3:**
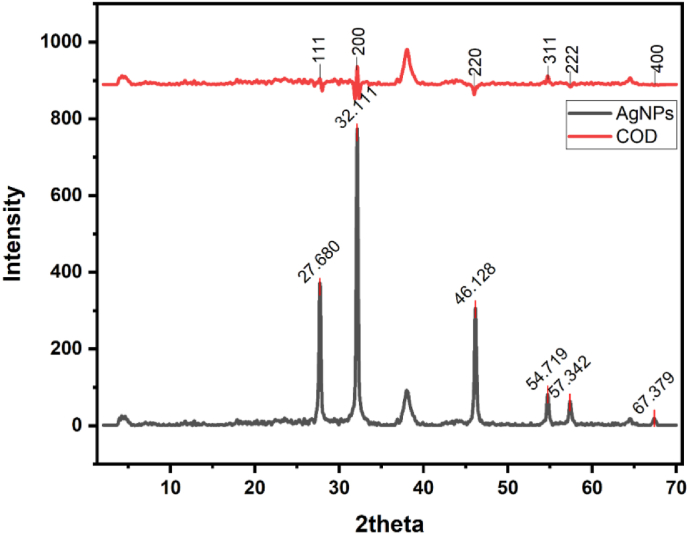
XRD patterns of the synthesized nanoparticles (the black line represents patterns of the AgNPs while the red represents a COD (crystallography open database) plane).

The crystallinity nature and phase comparison of the synthesized silver NPs utilizing *S. cuneifolia* leaf extract were examined through X-ray diffraction (XRD) Fig. 3. The diffraction pattern obtained, accompanied by a reference from the Crystallography Open Database (COD), is illustrated in Fig. 3. The distinct diffraction patterns at 2theta values of 27.680°, 32.111°, 46.128°, 54.719°, 57.342°, and 67.379° correspond to crystallographic planes (hkl) 111, 200, 220, 311, 222, and 400 and corroborated with standard Chlorargyrite (AgCl) (PDF: 96-901-1667) respectively Fig. 3. This result verifies that the silver nanoparticles with a face-centered cubic (fcc) crystal structure were produced. These XRD spectra were similar to the spectra of AgNPs synthesized using *Fusarium scirpi* by Rodríguez-Serrano et al.^43^ and Illanes Tormena et al.^44^ Furthermore, the XRD spectrum revealed extra unmatched peaks, which may be linked with the crystallization of bio-organic substances on the silver nanoparticles' exterior. The phytochemical components found in the possible extract of the impurity peak are correlated with the noise.^36^, ^45^, ^46^, ^47^

### 3.4 Transmission electron microscope (TEM) analysis

**Fig. 4:**
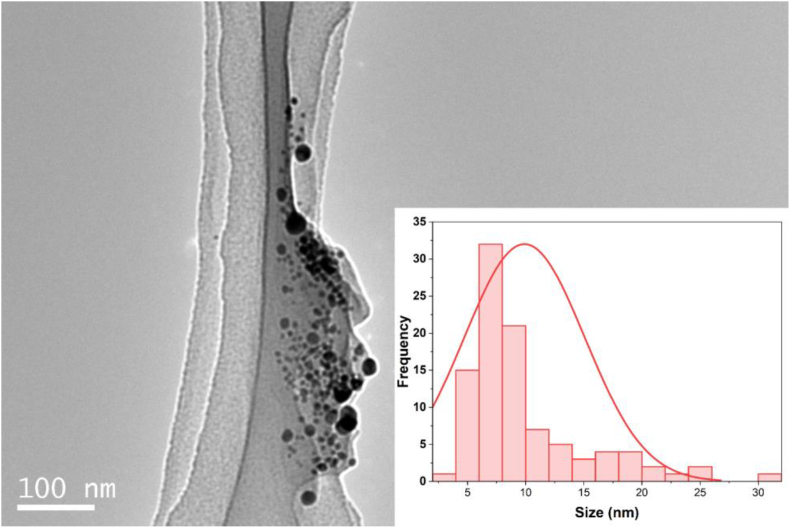
TEM image at 100 nm scale confirms the formation of nanoparticles with spherical shapes. Inset is a histogram representing the size distribution of *S. cuneifolia* extract-mediated AgNPs.

The critical data on the dimensions and morphological characteristics of silver nanoparticles synthesized utilizing *S. cuneifolia* aqueous leaf extract were discerned from the Transmission Electron Microscopy (TEM) image of the generated AgNPs Fig. 4. The produced particles exhibited a spherical morphology in nature, with diameters varying from 4 to 31 nm, non-aggregated, and extensively diffused across the surfaces which increases their durability and biological activity^41^, ^48^, ^49^ also essential for effective engagement with the envelope of the microbes and finally enhance the antimicrobial properties.^50^ ImageJ was used to calculate the sizes and particle dispersion from the TEM image^51^; the histogram graphical representation Fig. 4 of the generated Ag nanoparticles shows the NPs dispersion with average sizes of 9.9 ± 5.1 nm, which match the finding of other researchers synthesized AgNPs with sizes less than 100 nm.^18^, ^49^ TEM imaging further confirms the effective formation of nanoparticles utilizing *S. cuneifolia* leaf extract alongside UV–vis and XRD data.

### 3.5 Minimum inhibitory concentration and minimum bactericidal concentration

The plant extract showed no inhibitory effect towards all isolates at the highest testing concentration (32 mg/mL). While results in Table 2 show that the synthesized AgNPs demonstrated the capability to suppress the proliferation of *E. coli* (standard and (R)) and *S. aureus* (standard and (R)) strains equally, with the values of MIC recorded to be 31.25 μg/mL for each, and more superior against *S. flexneri* with the value of MIC of 15.62 μg/mL. However, the MBC results of the synthesized Ag nanoparticles mediated by *S. cuneifolia* showed their superiority towards *E. coli* and *S. flexneri* (Gram-negative), with MBC equal to the MIC, which were 31.25 μg/mL for both *E. coli* and 15.62 μg/mL for *S. flexneri* Table 2. Additionally, the MBC of Gram-positive *S. aureus* ATCC25923 was significantly higher at 125 μg/mL, indicating a lower bacteriostatic effect Table 2. At the highest tested concentration (500 μg/mL), the AgNPs exhibited no bactericidal activity against *S. aureus* (Resistant), which indicates higher resistance to Ag nanoparticles compared to the susceptible strain. This result aligns with the findings of *Kumari* et al., in which values of the MIC of plant extract-mediated AgNP synthesis against *E. coli* ranged from 25 to 50 μg/mL.^52^ Also, Vanlalveni et al. reported a significant difference between the MIC values of AgNPs using *Ocimum sanctum* extract for the susceptible *S. aureus* and resistant strains (Vanlalveni et al., 2021). Gram-positive bacteria's dense and stiff cellular envelope structures and Gram-negative bacteria's strong lipopolysaccharide membranes can be penetrated by the green-synthesized AgNPs mediated by *S. cuneifolia* extract, resulting from their diminutive size.^12^ The development of intracellular reactive oxygen species (ROS) is facilitated by the ionization of ingested AgNPs, which release silver ions (Ag^+^). Ultimately, these ROS induce cell demise by inflicting damage on the intracellular components, including nucleic acids (DNA and RNA), proteins, lipids, and other necessary components.^11^, ^47^, ^53^

**Table 2:** MIC and MBC of AgNPs synthesized using *S. cuneifolia* leaf extract.

Bacterial strain	MIC μg/mL	MBC μg/mL	MIC μg/mL		
	AgNPs		Ceftriaxone	Vancomycin	Amikacin
*E. coli* ATCC25922	31.25	31.25	≤0.48	–	1.9
*E. coli* (R)	31.25	31.25	>250	–	250
*S. flexneri* ATCC12522	15.62	15.62	≤0.48	–	7.8
*S. aureus* ATCC25923	31.25	125	1.9	7.8	–
*S. aureus* (R)	31.25	>500	62.5	15.62	–

### 3.6 Disc diffusion assay

**Fig. 5:**
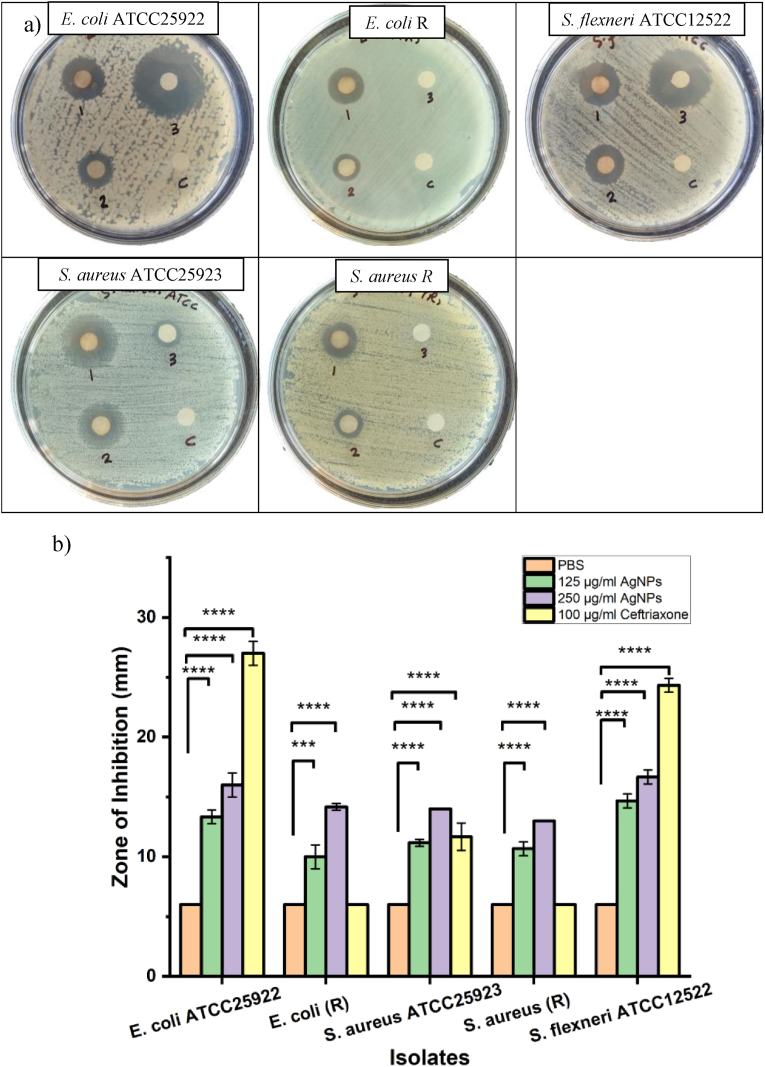
Antibacterial evaluation of synthesized AgNPs on tested bacteria. (a) The agar diffusion assay employed several pretreatment methods on *E. coli*, *S. flexneri*, and *S. aureus* (1: 5 μg/disc AgNPs, 2: 2.5 μg/disc AgNPs, 3: 100 μg/mL Ceftriaxone, and C: PBS). (b) Was the statistical analysis results of the diameter of the inhibition zone (mm) of (a), the diameters of the inhibition zone of AgNPs were significantly different (∗∗P ˂ 0.01, ∗∗∗P ˂ 0.001, ∗∗∗∗P ˂ 0.0001) compared to the negative control as well as at various concentration levels.

The disc diffusion assay was utilized to assess the in vitro antibacterial activity in addition to MIC of *S. cuneifolia*-mediated AgNPs synthesis against different bacterial strains, including *E. coli* (ATCC25922 and resistant), *S. flexneri* ATCC12522, and *S. aureus* (ATCC25923 and resistant) as illustrated in Fig. 5(a). As expected, none of the targeted bacterial strains showed inhibition by the negative control (PBS) group. The inhibitory zone was clearly observable across all strains subjected to silver nanoparticles at varying concentrations (2.5 and 5 μg/disc). The AgNPs in the 5 μg/disc group showed a higher inhibition zone than the 2.5 μg/disc concentration group, indicating a dose-dependent antibacterial effect. However, the only susceptible bacterial strains (*E. coli* ATCC25922, *S. flexneri* ATCC12522, and *S. aureus* ATCC25923) were suppressed by the 100 μg/mL Ceftriaxone (positive control), but none of the resistant strains (*E. coli* and *S. aureus*) Fig. 5(a). This demonstrates the antibacterial effect on susceptible and resistant strains (Gram-positive and -negative). The findings indicated that the AgNPs synthesized using *S. cuneifolia* leaf extract exhibited superior antibacterial properties, indicating their possible use as an innovative antimicrobial solution for effectively addressing bacterial infections.

Fig. 5(b) shows the diameter of the inhibition zone and the statistical analysis of the results of Fig. 5(a). As represented in Fig. 5(b), the zone of inhibition diameters (mm) of AgNPs against *E. coli* ATCC25922, *E. coli* (R), *S. flexneri* ATCC12522, *S. aureus* ATCC25923, and *S. aureus* (R) at a concentration of 5 μg/dis are 16 ± 1, 14.1 ± 0.2, 16.6 ± 0.5, 14, and 13 mm, and at a concentration of 2.5 μg/disc are 13.3 ± 0.5, 10 ± 1, 14.6 ± 0.5, 11.1 ± 0.2, and 10.6 ± 0.5, respectively. Compared to the PBS control group, the diameters of the zone of inhibition (mm) were significantly different against the tested bacteria and at the concentration variance (∗P < 0.05,∗∗P < 0.01,∗∗∗P ˂ 0.001,∗∗∗∗P ˂ 0.0001). In contrast, the zone of inhibition (mm) of the positive control (Ceftriaxone) was 27 ± 1, 0, 24.3 ± 0.5, 11.6 ± 1.1, and 0 mm against *E. coli* ATCC25922, *E. coli* (R), *S. flexneri* ATCC12522, *S. aureus* ATCC25923, and *S. aureus* (R), respectively. Notably, it is visible that the inhibition zones of the Ag nanoparticle synthesized using *S. cuneifolia* extract against targeted strains were highly susceptible to *E. coli* and *S. flexneri* (Gram-negative) compared to *S. aureus* (Gram-positive). This susceptibility difference may be attributed to the difference in the bacteria's envelope structure (cell wall),^54^ as the thickness of the peptidoglycan layer is thinner in Gram-negative bacteria, which may facilitate better penetration of nanoparticles.^10^, ^55^ The scientists discovered that the AgNPs firmly attach to the cell wall of bacteria and infiltrate the cells, causing cellular demise by compromising the cell membrane.^12^ K. S. Allemailem et al.,^56^ reported that the AgNPs synthesized using Ajwa date extract (Aw-AgNPs) at a concentration of 100 μg/mL exhibited notable antimicrobial properties towards Gram-positive and –negative bacteria with significant anti-biofilm properties, which is comparable to this study.

### 3.7 Mode of action

**Fig. 6:**
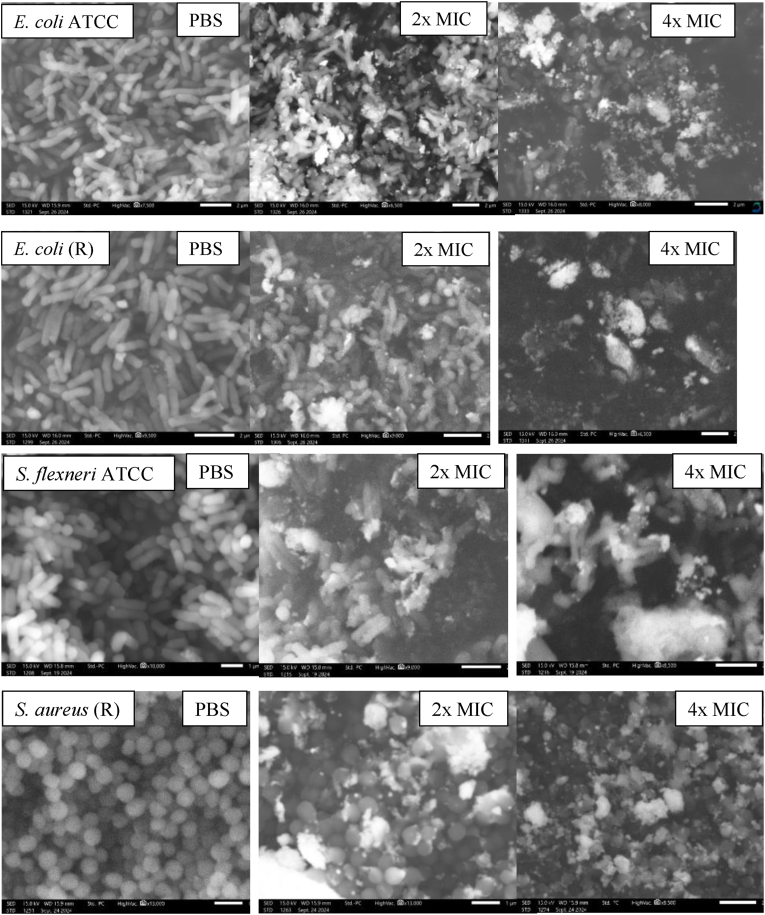
SEM images of the morphological characterization of *E. coli* (susceptible and resistant), *S. flexneri*, and *S. aureus* treated with different concentrations (2× and 4× MIC) of AgNPs.

SEM was further used to investigate the effect of the synthesized AgNPs on the morphological structure of the selected strains of bacteria, including *E. coli* (susceptible and resistant), *S. aureus* resistance, and *S. flexneri* treated with 2× and 4× MIC for an hour, and PBS was used as a negative control. As shown in Fig. 6 the cells exhibit a smooth morphological structure in the PBS-treated bacteria, which becomes compromised upon treatment with AgNPs in a dose-dependent manner. However, the 2× MIC doses induce mostly cellular shrinkage and irregularities on the cell membrane and less cell destruction. In contrast, the 4× MIC dose showed total membrane integrity failure, leading to the discharge of intracellular contents as in the *E. coli* (R) or more ruptured cells than shrunk ones as in *E. coli* ATCC25922, *S. flexneri*, and *S. aureus* (R). The observed alterations in cell morphology indicate that the synthesized AgNPs induce bacteriostasis or bactericidal effects through the breakdown of the cell membrane. The elevated responsiveness and bonding effectiveness towards protein molecules, achieved through modifying the bacterial cell's and nuclear membrane's structure, leads to cellular disruption and destruction. Alongside their ability to damage the bacterial membrane, AgNPs are recognized for their antimicrobial effects, which operate through various mechanisms. A significant mechanism includes the production of reactive oxygen species (ROS), which may result in oxidative stress, causing harm to cellular elements like proteins, lipids, and nucleic acids.^57^ Moreover, it has been documented that AgNPs directly engage with the DNA of the bacteria, hindering the processes of replication and transcription, while also binding to other molecules such as proteins, which may lead to changes in the function of the enzyme and disruption of metabolism processes.^58^, ^59^ The interplay of these mechanisms likely underpins the strong antibacterial and anti-biofilm effects noted in our findings. Silver nanoparticles (AgNPs) measuring 10–100 nm demonstrated significant bactericidal efficacy towards both gram-positive and -negative bacteria.^60^ Modifications in the structure of bacterial cells and nuclear membranes accompany the elevated responsiveness and binding effectiveness with proteins. Ultimately, this leads to cellular disruption and mortality. Silver ions possess the capacity to impede replication of bacteria by attaching to and denaturing the DNA of bacteria, a process that involves how proteins' thiol groups interact with silver ions, leading to DNA condensation and subsequent apoptosis.^25^ AgNPs can readily infiltrate the microbial cell wall owing to their smaller size compared to bacteria. Silver nanoparticles (AgNPs) are employed in the food sector for packaging to prevent food product contamination by bacteria.^25^

### 3.8 Hemolysis assay

**Fig. 7:**
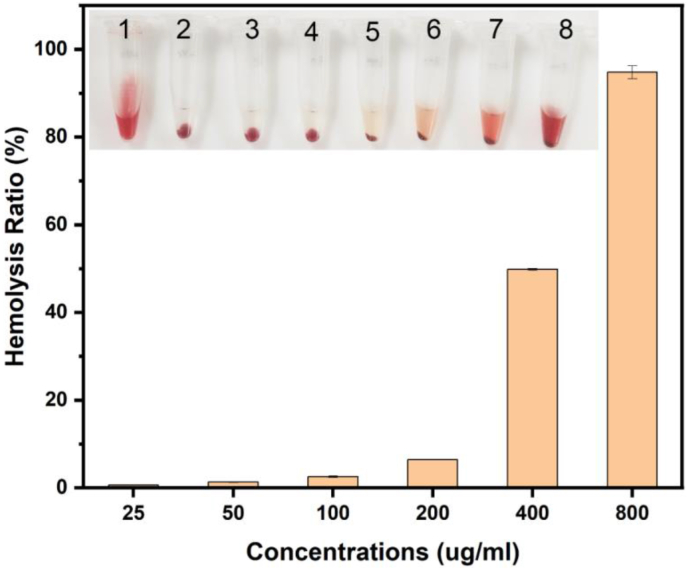
The hemolysis ratio of the synthesized AgNPs using *S. cuneifolia* leaf extract. The inset displays a digital image of hemolysis experiments subjected to different concentrations (1: 1 % positive control, 2: negative control, and 3–8 are 25–800 μg/mL (two-fold dilution)).

For practical use, it is essential that nanoparticles possess exceptional antibacterial efficacy while demonstrating low toxicity.^61^ Higher concentrations of nanoparticles can harm organisms^62^ Thus, the hemolytic activity of the Ag nanoparticles was assessed to determine biosafety. As shown in Fig. 7 the concentration of synthesized nanoparticles up to 100 μg/mL showed an excellent hemolysis ratio, which was 2.55 %, and the hemolysis ratio increased slightly to 6.42 % at the concentration of 200 μg/mL, which is still within the accepted rate (below 10 %).^63^ At 400 μg/mL concentrations and 800 μg/mL, the hemolysis ratio of the AgNPs increased notably, which were 49.83 % and 94.75 %, respectively Fig. 7. Xu et al. reported that the silver nanocomposite exhibited a hemolysis rate of less than 5 % at the concentration of 100 μg/mL.^64^ In this research, AgNPs at 100 μg/mL demonstrated a hemolysis ratio of less than 5 %, which is considered safe according to ISO/TR 7406 and various scientific studies.^65^ Additionally, this dose effectively inhibited the planktonic bacterial growth as well as the formation of biofilms, indicating an appropriate balance between biological compatibility and efficacy. Similar research has shown that AgNPs at concentrations below 100 μg/mL exhibit potent antibacterial properties without harming human erythrocytes or other primary cell lines.^66^, ^67^ Khadri et al.,^68^ reported that 10 mg/mL was the minimum concentration of AgNPs to show cytotoxicity (IC_50_) against the cell line. Although in vitro results are encouraging, additional animal model studies would be necessary to confirm therapeutic applicability.

### 3.9 Anti-biofilm evaluation

**Fig. 8:**
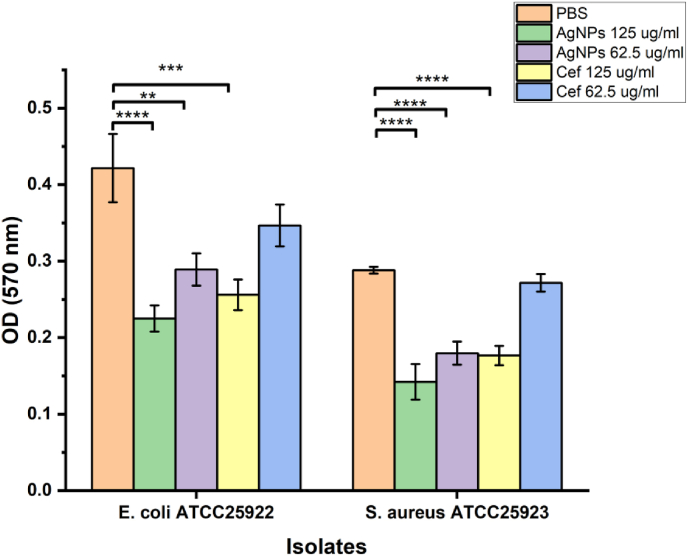
Anti-biofilm evaluation of AgNPs and positive control Ceftriaxone against *S. aureus* ATCC25923 and *E. coli* ATCC25922 using semi-quantitative analysis of crystal violet staining, the destruction of the biofilm strains was significantly different (∗∗P < 0.01, ∗∗∗P < 0.001, ∗∗∗∗P < 0.0001) compared to control (PBS).

The semi-quantitative analysis of crystal violet was used to evaluate the effect of bio-synthesized AgNPs using *S. cuneifolia* leaf extract against biofilm formation by *S. aureus* ATCC25923 and *E. coli* ATCC25922 bacterial strains. It has been found that the concentration of 5 × 10^7^ CFU/mL of the bacteria was the most suitable concentration for the biofilm biomass formation,^64^ which was used in this study. The biofilm destruction was assessed in a dose-dependent manner; however, the AgNPs slightly destroyed the biofilms formed by Gram-negative *E. coli* at the concentration of 62.5 μg/mL and the destructions increased with the increase of the concentration Fig. 8. In contrast, the destruction of the developed biofilm biomasses by the Gram-positive *S. aureus* was destroyed significantly at all the different concentration levels (62.5 and 125 μg/mL) Fig. 8. As shown in Fig. 8 the destruction of the Gram-positive and -negative biofilms by the AgNPs were significantly different compared to the PBS-treated control group and at concentration difference as well (∗∗P < 0.01, and ∗∗∗P < 0.001). On the other hand, the positive control, ceftriaxone, destroyed the biofilms significantly at higher concentrations only (125 μg/mL). Furthermore, the biofilm inhibition % was determined, as shown in Table 3 at a concentration of 125 μg/mL, AgNPs a 46.64 % and 50.58 % inhibition rate against *E. coli* and *S. aureus* respectively, which exceeds the activity noted with the same concentration of ceftriaxone which was 39.29 % and 38.66 % towards *E. coli* and *S. aureus* respectively. At a concentration of 62.5 μg/mL, AgNPs exhibited significant anti-biofilm activity, surpassing the effectiveness of the equivalent lower dose of ceftriaxone. As reported by Kaliappan et al.,^69^ CA-AgNPs demonstrated up to 82 % and 79 % biofilm inhibition toward *E. coli* and *S. aureus,* respectively, which aligns with our findings that the biofilm destruction by AgNPs increases with the increment of the concentration. Biofilm is known as one of the resistance mechanisms that acts as a shield to protect bacterial cells from antimicrobial agents.^70^ The mechanisms behind the destruction of biofilms by silver nanoparticles involve the interaction of positively charged AgNPs with the negatively charged components of EPS (extracellular polymeric substances), which enables AgNPs to adhere to and penetrate the biofilm matrix, leading to the degradation of polysaccharides and proteins within the EPS. Finally, it weakens the structure of the biofilms, making the bacteria more susceptible.^14^, ^71^ Many studies have reported that nanoparticles, due to their smaller sizes, exhibit better diffusion through the biofilm matrix.^71^

**Table 3:** Biofilm inhibition % of AgNPs and ceftriaxone at different concentrations.

Treatment μg/mL		Biofilm inhibition %	
		*E. coli* ATCC25922	*S. aureus* ATCC25923
AgNPs	125 μg/mL	46.64 %	50.58 %
	62.5 μg/mL	31.46 %	37.62 %
Ceftriaxone	125 μg/mL	39.29 %	38.66 %
	62.5 μg/mL	17.79 %	5.67 %

### 3.10 Long-term storage antimicrobial evaluation of the AgNPs

**Fig. 9:**
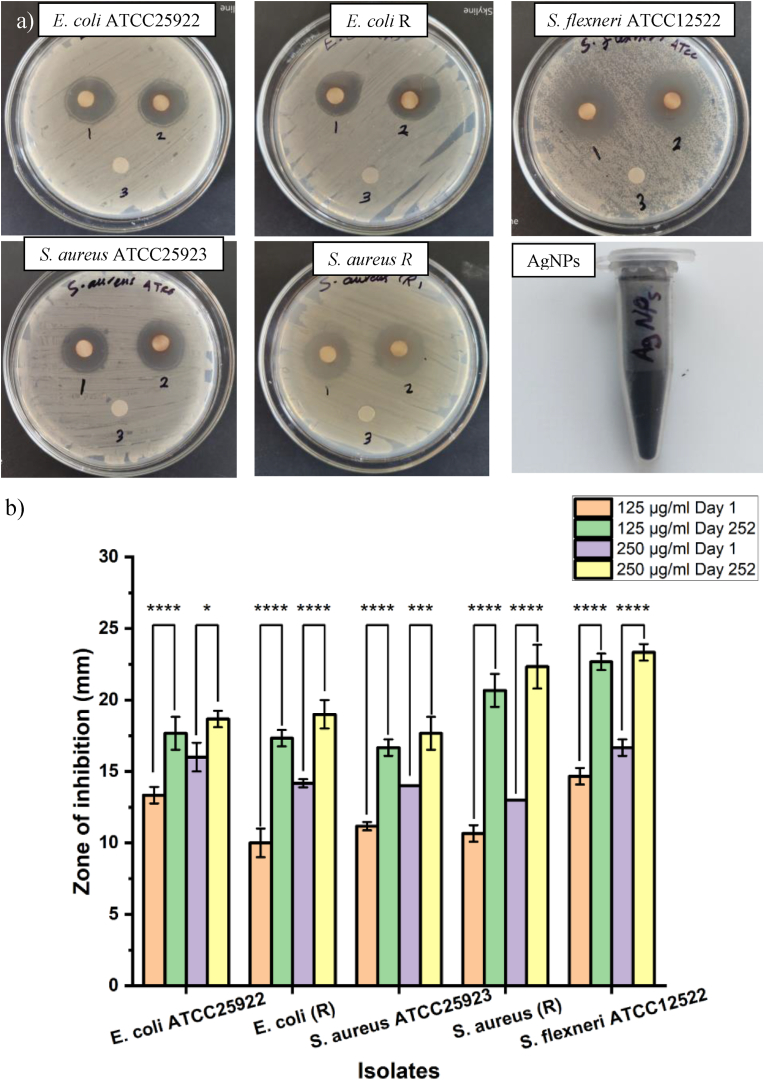
Antibacterial evaluation of synthesized AgNPs on tested bacteria after 252 days and lyophilized preserved stock of the AgNPs. (a) The agar diffusion assay employed two different concentrations of *E. coli*, *S. flexneri*, and *S. aureus* (1: 2.5 μg/disc AgNPs, 2: 5 μg/disc AgNPs, 3: PBS). (b) The statistical analysis results for the diameter of the inhibition zone (mm) of AgNPs at day 1, compared to zones after 252 days of storage. At the level of 0.05, results were significantly different, where (∗P < 0.05, ∗∗P ˂ 0.01, ∗∗∗P ˂ 0.001, ∗∗∗∗P ˂ 0.0001).

The disc diffusion assay was utilized to assess the antibacterial activity of long-term storage of lyophilized AgNPs against the tested bacteria. At the concentration of 2.5 μg/disc AgNPs, zones of inhibitions were 17.6 ± 1.1, 17.3 ± 0.5, 16.6 ± 0.5, 20.6 ± 1.1, and 22.6 ± 1.1 mm corresponding to *E. coli* ATCC25922, *E. coli* (R), *S. aureus* ATCC25923, *S. aureus* (R), and *S. flexneri* ATCC12522, respectively, and at 5 μg/disc zones were 18.6 ± 1.1, 19 ± 1, 17.6 ± 1.1, 22.3 ± 1.5, and 23.3 ± 0.5 mm corresponding to *E. coli* ATCC25922, *E. coli* (R), *S. aureus* ATCC25923, *S. aureus* (R), and *S. flexneri* ATCC12522, respectively as illustrated in Fig. 9(a). Fig. 9(b), showing the diameter of the inhibition zone of the results of Fig. 9(a) and the statistical analysis of the zone of inhibition at day 1, Fig. 5(a), compared to the zones of inhibition of the AgNPs after long-term storage (8 months plus), Fig. 9(a). As the results showed, the long-term storage of the AgNPs in a powder form at room temperature does not affect the antimicrobial properties. The antibacterial properties against both Gram-positive and Gram-negative bacteria have been significantly increased after storage compared to those when it was freshly prepared. Diameters of the zone of inhibition were significantly different against the tested bacteria before and after storage (∗P˂0.05, ∗∗P < 0.01,∗∗∗P < 0.001,∗∗∗∗P < 0.0001). This investigation demonstrated that the lyophilized AgNPs exhibit markedly greater stability than liquid suspensions, as dispersion—particularly in the presence of oxygen, light, or moisture—can expedite aggregation and oxidation during storage.^28^, ^29^ For example, Korshed et al.^28^ reported that the antibacterial efficacy of AgNPs in suspension form increased significantly after 112–140 days of storage compared to 14 days, and the nanoparticles began to lose their activity after this time, until they completely lost their activity after 260–280 days of storage. Another study reported that the AgNPs started losing their antimicrobial effectiveness after 90 days of storage in colloidal form.^29^ Compared to this study, in which the antimicrobial efficacy was retained at a higher level after 252 days, suggesting that the best way of AgNPs storage is in powder form.

## 4 Conclusion

Due to the globalization of AMR and the failure of traditional antibiotics, nanotechnology could be a better alternative to antibiotics for AMR. This study, presenting a straightforward and environmentally friendly method for synthesizing AgNPs using S. cuneifolia leaf extract, promoting sustainable synthesis. The use of *S. cuneifolia* leaf extract functions as a reducing agent, stabilizing agent, and capping agent, therefore promoting the synthesis of stable Ag nanoparticles. Changes in the color and UV–vis spectrum confirmed AgNPs formation with a distinct peak at 419 nm. X-ray diffraction patterns verify that the AgNPs have a face-centered cubic (fcc) crystal structure at 2theta degree, the TEM further confirmed that they are primarily spherical in morphology with sizes ranging from 14 to 78 nm, and FTIR spectroscopy examination indicates significant vibrations of O–H, C–H, C=O, C–N, C–H, C–N stretching, and C–O stretching. Additionally, the fabricated Ag nanoparticles exhibit a notable antibacterial effect in a dose-dependent manner at (5 and 2.5 μg/disc) against Gram-negative and -positive bacteria with significant differences compared to the control (∗∗P ˂ 0.01,∗∗∗P ˂ 0.001,∗∗∗∗P ˂ 0.0001). The MICs are determined to be 31.25 μg/mL for each *E. coli* and *S. aureus*. The MBC of AgNPs against Gram-negative bacteria was equal to their MIC, while it was higher against Gram-positive bacteria, and 15.62 μg/mL for *S. flexneri*. The MBC of AgNPs against Gram-negative bacteria was equal to their MIC, while the MBC against Gram-positive bacteria was higher. Furthermore, SEM has confirmed that the AgNPs are acting on the cell membrane, causing damage and leakage of the interior molecules. Moreover, the synthesized nanoparticles reveal a significant anti-biofilm effect against Gram-positive and -negative biofilms. Additionally, the results demonstrated that AgNPs exhibit safe biocompatibility at 100 μg/mL, as indicated by the absence of hemolysis. Thus, the leaf extract from *S. cuneifolia* appears to hold potential as a sustainable, eco-friendly alternative chemical method for Ag nanoparticle synthesis, exhibiting antibacterial and anti-biofilm activities with low cytotoxicity. The *S. cuneifolia* leaf extract-mediated green synthesis of AgNPs exhibits antibacterial and anti-biofilm properties. It is anticipated to be developed into an antibacterial and anti-biofilm agent to address antimicrobial resistance issues.

## CRediT authorship contribution statement

**Motasim Ismael:** Writing – review & editing, Writing – original draft, Visualization, Validation, Software, Resources, Methodology, Investigation, Formal analysis, Data curation, Conceptualization. **Madivoli Edwin:** Writing – review & editing, Supervision, Conceptualization. **Khayeli Juliah:** Writing – review & editing, Supervision.

## Funding

This research was funded by the African Union under its scholarship program through the Pan African University Institute for Basic Sciences, Technology, and Innovation (PAUSTI).

## Declaration of competing interest

We confirm that this manuscript is original, has not been published elsewhere, and is not under consideration by another journal. All authors have approved the manuscript and have no conflicts of interest to disclose.
