# Supplementary material for: Biogenic synthesis of silver nanoparticles using Sida cuneifolia leaf extract for enhanced antibacterial, cytotoxic, and anti-biofilm activities
Source: Biotechnol Notes. 2025 Jul 28;6:196–208. doi: 10.1016/j.biotno.2025.07.003 (PMC12365124; doi:10.1016/j.biotno.2025.07.003)
Supplement: Multimedia component 1 [file mmc1.pdf]

Mount Kenya University

REF: **MKU/ISERC/4066** Date: 31 July 2024

TO: **Motasim Noureldin Mohammed Ismael**

REG: **MB400-0002/202**

Dear Sir/Madam,

RE: Development and evaluation of silver nanoparticles-based synthetic peptides inspired by pep6 against multidrug-resistant bacteria (E. coli and S. aureus)

This is to inform you that **Mount Kenya University** has reviewed and approved your above research proposal. Your application approval number is **2960.** The approval period is **31/07/2024 - 30/07/2025.**

This approval is subject to compliance with the following requirements;

i. Only approved documents including informed consents, study instruments, MTA will be used
ii. All changes including amendments, deviations and violations are submitted for review and approval by **Mount Kenya University**
iii. Death and life-threatening problems and serious adverse events or unexpected adverse events whether related or unrelated to the study must be reported to **Mount Kenya University** within 72 hours of notification
iv. Any changes, anticipated or otherwise that may increase the risks or affect the safety or welfare of study participants and others or affect the integrity of the research must be reported to **Mount Kenya University** within 72 hours
v. Clearance for export of biological specimens must be obtained from relevant institutions
vi. Submission of a request for renewal of approval at least 60 days prior to expiry of the approval period. Attach a comprehensive progress report to support the renewal
vii. Submission of an executive summary report within 90 days upon completion of the study to **Mount Kenya University**

Prior to commencing your study, you will be expected to obtain a research license from National Commission for Science, Technology and Innovation (NACOSTI) https://research-portal.nacosti.go.ke and also obtain other clearances needed.

Yours sincerely,

MOUNT KENYA UNIVERSITY
ETHICS REVIEW COMMITTEE
P. O. Box 342 - 01000,
THIKA

Dr. Alfred Owino, PhD
Chairman, Mount Kenya University ISERC
